# On the evolving portfolio of community-standards and data sharing policies: turning challenges into new opportunities

**DOI:** 10.1186/2047-217X-1-10

**Published:** 2012-07-12

**Authors:** Susanna-Assunta Sansone, Philippe Rocca-Serra

**Affiliations:** 1 Oxford e-Research Centre, University of Oxford, Oxford, UK

**Keywords:** Standard, Ontology, Data sharing, Curation, Data policy, ISA commons, ISA-Tab, BioSharing

## Abstract

There are thousands of biology databases with hundreds of terminologies, reporting guidelines, representations models, and exchange formats to help annotate, report, and share bioscience investigations. It is evident, however, that researchers and bioinformaticians struggle to navigate the various standards and to find the appropriate database to collect, manage, and share data. Further, policy makers, funders, and publishers lack sufficient information to formulate their guidelines. In this paper, we highlight a number of key issues that can be used to turn these challenges into new opportunities. It is time for all stakeholders to work together to reconcile cause and effect and make the data-sharing culture functional and efficient.

## Wealth of data sharing enablers: yet still a challenging practice

Shared, annotated bioscience research data and methods offer new discovery opportunities and prevent unnecessary repetition of work. In the last decade, several data preservation, management, sharing policies, and plans have emerged in response to increased funding for high-throughput approaches in genomics and functional genomics science [1]. In parallel, a growing number of community-based groups have developed minimum information requirements, terminologies, models, and exchange formats to standardize their system of reporting different experiments [2], and they have worked to maximize the interoperability among these standards [3,4]. Researchers and bioinformaticians in both academic and commercial science [5], along with funding agencies and publishers, embrace the concept that standards are pivotal to enriching the annotation of the entities of interest (e.g., genes, metabolites) and the experimental steps (e.g., provenance of study materials, technology and measurement types), to ensure that shared investigations are comprehensible and (in principle) reproducible.

As a consequence of this ‘general mobilization’, there are thousands of biology databases, over 300 terminologies, and more than 150 reporting guidelines, representations models, and exchange formats that are meant to help with bioscience annotation, reporting, and sharing. But how many times have you asked or have been asked questions, like: “*I work with stem cells*, *which terminologies are applicable to my domain*?” “*Are there standards and tools for publishing and archiving my* (*meta*)*genomics and* (*meta*)*transcriptomics experiments*? *If not*, *what are the steps and methods to mobilize the community and develop these collaboratively*?”, “*My funding agency's data sharing policy recommends the use of 'established, community standards', but which ones are widely endorsed and applicable to my wheat functional genomics data*?”. This inquisitive and explorative attitude is a tangible sign of the positive effect of the growing movement for producing easily reproducible research. But, although, most stakeholder groups accept the principles of proactive data sharing, compliance is challenging in practice. Vast swathes of bioscience data still remain locked in esoteric formats, are described using *ad hoc* or proprietary terminology, or lack sufficient contextual information; many tools do not implement standards — even where these exists. But what are the reasons behind this?

Recently, a session on data policy and standards, organized at the annual Genomic Standards Consortium meeting (BGI, March 2012), provided a snapshot of the current situation [6]. We highlight here a number of key issues that emerged, enriched by our experiences over the last decade by working with a variety of stakeholders, including researchers, bioinformaticians, developers in public and private sectors, standards-developing communities, as well as funders and publishers.

## Evidence-informed guidance life cycle: the chicken and egg conundrum

***Publishers*** and ***funders*** do strive to play an active role. *Nature Biotechnology*, for example, has published over 20 papers on standards, often after soliciting an open review by the broader community; BioMed Central leads a ‘Publishing Open Data Working Group’ [7] to stimulate debate among authors, publishers, funders, and librarians to identify mutually agreeable ways for implementing data sharing/deposition policies. Funding agencies work to collect community views and feedback by issuing on-going ‘Requests for Information’. But despite these clearly positive signs, their guidance (to authors, applicants and awardees) is not always anchored on solid ground, primarily because they often do not have enough evidence to make informed decisions on which standards for data sharing resources should be recommended. Consequently, their guidance text is often loosely formed, and basically suggests use of “*recognised standards*”, where these exist, and make data available through existing community resources or databases “*where possible*”. This again highlights the lack of broadly agreed upon policies for emerging data types. A similar scenario is found in the publishing world, where a substantial proportion of original research papers published in high-impact journals are either not subject to any data availability policies or do not adhere to the data availability instructions in their respective journals [8].

***Researchers***, ***bioinformaticians*,** and ***developers*** also lack support and are left to deal with the interpretation of data sharing policies to the best of their knowledge. They are also not always equipped to navigate and select the most appropriate standards, among the dearth of the domain-specific offerings, and end up seeing standards as burdensome and over-prescriptive. This is only furthered aggravated because tools/databases have not managed to enable their ‘invisible use’, as it should be — but, to be fair, this is not a trivial task. The mountain of technical frameworks needed to implement a standard, or multiple standards, inhibits the development of standard-compliant tools and databases, hence their adoption.

## Every challenge is an opportunity: let’s roll up our sleeves

The cost of implementing a standards-supported data sharing vision is as large as the number of stakeholders who must operate synchronously. The extensive ‘*social engineering*’ and *community liaison* need to be managed and funded, and *rewards* and *incentives* need to be identified for all contributors in the development and implementation of standards. The *stakeholders*’ *communication* is naturally organic, but unfortunately this also means it is quite patchy and *ad hoc*. We need to nurture an open, integrative, and pre-competitive communication environment that connects all parties during the development and evolution of standards and policies, but that also cultivates the collective expertise and experience, recording invaluable *feedback cycles*, and facilitating the complex unpacking *stakeholders*’ *dynamics*, where it can be refined and used to inform the next steps.

*Ownership* of open standards can be problematic in broad, grass-roots collaborations; the embryonic *legal framework* in this area requires new or improved models to encourage maintenance of and contribution to open standards and support their evolution. Only rarely are appropriate *funding mechanisms* provided to support such a large, time consuming, mainly volunteer-based, undertaking. Robust relationships among all stakeholders can help to ensure a *long*-*term sustainability* strategy for these endeavours, where the costs will further accrue as the standards or the tools are refined, adopted, and evolve to serve new data type and users’ needs. When funds are mobilized, budgetary constraints will also require our building a comprehensive picture of the current *portfolio of data sharing enablers* to make sure that those areas that are in greatest need are addressed, harmonization is encouraged, and wasteful reinvention is ended.

When a standard is mature and appropriate standard-compliant systems become available, these then must be channelled to the appropriate stakeholder community, who in turn must use them to facilitate a high-quality data cycle, from data generation to standardization, and through publication to subsequent sharing and reuse. They also need to either endorse and require them in the data policies and begin to actively monitor adherence.

Although daunting, potential solutions to these issues are in fact within our reach, and thus provide an opportunity to create new relationships and collaborative models. Here are two examples: First, BioSharing [2], which works as a registry for community-standards, allies with the International Society for Biocuration and several other existing resources’ portals and catalogues. As such it creates common metadata descriptors to best categorize data sharing resources and builds a distributed ecosystem of inter-connected resources [9]. Second, the ISA Commons, which illustrates how the synergy between research and service groups, across a variety of life science domains, can work to build an network of data collection, curation, and sharing solutions that progressively enable the ‘invisible use’ of standards [10].

At this time, however, this remains a drop in the ocean; to achieve these goals all stakeholders must play their part. The real impact of standards and their economical value will be measured as we continue to facilitate their usability to improve data sharing and will demonstrate how this, in turn, underpins new biological insights and drives science of the future.

## Competing interests

The authors declared that they don’t have any competing interests.

## Authors’ contributions

SAS wrote the first draft with input from PRS, based on their experience over the last decade. All authors have read and approved the final manuscript.
